# Targeting papain-like protease for broad-spectrum coronavirus inhibition

**DOI:** 10.1007/s13238-022-00909-3

**Published:** 2022-04-06

**Authors:** Shuofeng Yuan, Xiaopan Gao, Kaiming Tang, Jian-Piao Cai, Menglong Hu, Peng Luo, Lei Wen, Zi-Wei Ye, Cuiting Luo, Jessica Oi-Ling Tsang, Chris Chun-Yiu Chan, Yaoqiang Huang, Jianli Cao, Ronghui Liang, Zhenzhi Qin, Bo Qin, Feifei Yin, Hin Chu, Dong-Yan Jin, Ren Sun, Jasper Fuk-Woo Chan, Sheng Cui, Kwok-Yung Yuen

**Affiliations:** 1 State Key Laboratory of Emerging Infectious Diseases, Li Ka Shing Faculty of Medicine, The University of Hong Kong, Pokfulam, Hong Kong SAR China; 2 Department of Microbiology, Li Ka Shing, Faculty of Medicine, The University of Hong Kong, Pokfulam, Hong Kong SAR China; 3 NHC Key Laboratory of Systems Biology of Pathogens, Institute of Pathogen Biology, Chinese Academy of Medical Sciences and Peking Union Medical College, Beijing, 100730 China; 4 School of Biomedical Sciences, Li Ka Shing Faculty of Medicine, The University of Hong Kong, Pokfulam, Hong Kong SAR China; 5 Department of Molecular and Medical Pharmacology, David Geffen School of Medicine, University of California Los Angeles, Los Angeles, CA USA; 6 Key Laboratory of Tropical Translational Medicine of Ministry of Education, Hainan Medical University, Haikou, 571199 China; 7 Academician Workstation of Hainan Province, Hainan Medical University, Haikou, 571199 China; 8 Hainan Medical University-The University of Hong Kong Joint Laboratory of Tropical Infectious Diseases, The University of Hong Kong, Pokfulam, Hong Kong Special Administrative Region China; 9 Guangzhou Laboratory, Guangzhou, 510320 China; 10 Department of Clinical Microbiology and Infection Control, The University of Hong Kong-Shenzhen Hospital, Shenzhen, 518053 China

**Keywords:** protease, inhibitor, coronavirus, Nsp3, antiviral

## Abstract

The emergence of SARS-CoV-2 variants of concern and repeated outbreaks of coronavirus epidemics in the past two decades emphasize the need for next-generation pan-coronaviral therapeutics. Drugging the multi-functional papain-like protease (PLpro) domain of the viral nsp3 holds promise. However, none of the known coronavirus PLpro inhibitors has been shown to be *in vivo* active. Herein, we screened a structurally diverse library of 50,080 compounds for potential coronavirus PLpro inhibitors and identified a noncovalent lead inhibitor F0213 that has broad-spectrum anti-coronaviral activity, including against the *Sarbecoviruses* (SARS-CoV-1 and SARS-CoV-2), *Merbecovirus* (MERS-CoV), as well as the *Alphacoronavirus* (hCoV-229E and hCoV-OC43). Importantly, F0213 confers protection in both SARS-CoV-2-infected hamsters and MERS-CoV-infected human DPP4-knockin mice. F0213 possesses a dual therapeutic functionality that suppresses coronavirus replication via blocking viral polyprotein cleavage, as well as promoting antiviral immunity by antagonizing the PLpro deubiquitinase activity. Despite the significant difference of substrate recognition, mode of inhibition studies suggest that F0213 is a competitive inhibitor against SARS2-PLpro via binding with the 157K amino acid residue, whereas an allosteric inhibitor of MERS-PLpro interacting with its 271E position. Our proof-of-concept findings demonstrated that PLpro is a valid target for the development of broad-spectrum anti-coronavirus agents. The orally administered F0213 may serve as a promising lead compound for combating the ongoing COVID-19 pandemic and future coronavirus outbreaks.

**Supplementary Information:**

The online version contains supplementary material available at 10.1007/s13238-022-00909-3.

## Introduction

The ongoing prevalence of COVID-19 and appearance of variants of concern (VOC) with more rapid transmission capacity, immune evasion has highlighted the general lack of antiviral small molecule drugs to fight a global coronavirus pandemic. Within the past two decades, another two coronaviruses have emerged to cause epidemics or pandemics in humans: severe acute respiratory syndrome coronavirus (SARS-CoV) in 2002–2003 and Middle East respiratory syndrome coronavirus (MERS-CoV) since 2012. Moreover, circulating common cold human coronaviruses (hCoV) including hCoV-OC43, hCoV-229E, hCoV-NL63, and hCoV-HKU1 contribute notably to morbidity, especially in the elderly and immunocompromised (Chan et al. 2015). Therefore, a highly effective antiviral with broad-spectrum coronaviruses coverage would facilitate the control of existing and emerging coronavirus diseases in the future.

Drugging the proteolytic viral PLpro are promising because coronaviruses expressing their protein machinery as a polyprotein that requires cleavage into functional units. Other PLpro enzymatic activities involve the removal of the cellular substrates ubiquitin (Ub), termed deubiquitination (DUB); and interferon-stimulated gene 15 (ISG15), termed deISGylation. Both DUB and deISGylation can cause viral evasion of the innate immune response (Barretto et al. 2005). As a result, coronaviruses with blocked PLpro protease activity lose their ability to replicate in cells. Strategically, targeting PLpro with antiviral drugs may have an advantage in not only inhibiting viral replication but also inhibiting the dysregulation of signaling cascades in infected cells that may lead to cell death in surrounding, uninfected cells (Klemm et al. 2020). PLpro is an essential part of the multi-domain nsp3 that constitutes a molecular pore across both membranes of the virus-induced double-membrane vesicles (DMV). While the viral replication complexes and viral RNAs are sealed in the DMVs, the nsp3 pore complex serve as a gateway for exporting viral RNA into cytoplasm (Wolff et al. 2020). Therefore, the unique subcellular localization of PLpro suggests it is a more accessible drug target than other nonstructural proteins sealed inside the DMVs.

**Figure 1:**
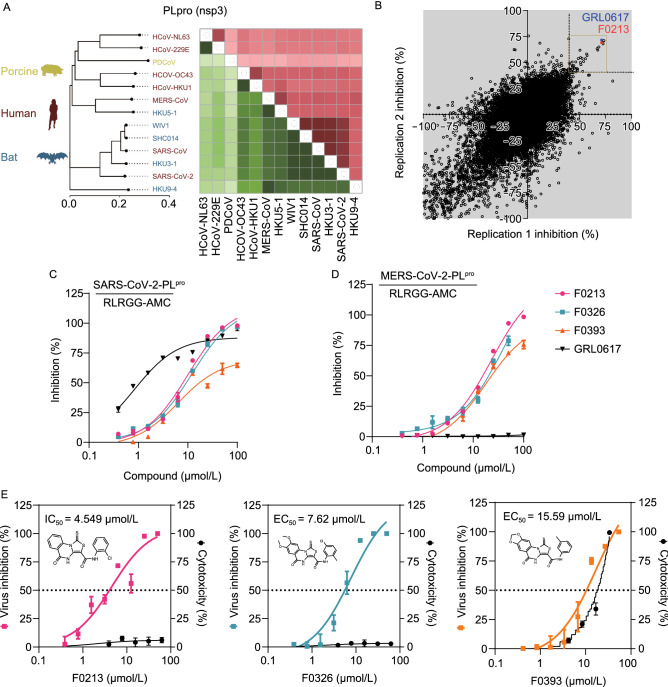
**High-throughput screening identifies dual inhibitors against SARS2-PLpro and MERS-PLpro**. (A) Neighbor-joining trees created with representative strains from all four CoV genogroups showing the genetic similarity and BLOSUM62 score of CoV-PLpro. Text color of the virus strain label corresponds to the susceptible host species on the left. The heat map adjacent to each neighbor-joining tree depicts percent amino acid similarity and BLOcks SUbstitution Matrix 62 (BLOSUM62 score) that indicating evolutionarily divergence. (B) Results from screening 50,080 compounds in duplicate for inhibition of PLpro activity. The replicate plot shows the percentage inhibition of PLpro by each compound. The structure of the lead compound F0213 is shown, and its activity is plotted in red. The hit zone for the assay (40% inhibition) is indicated by a white box. (C) *In vitro* inhibition for SARS2-PLpro of the selected primary hits. (D) *In vitro* inhibition for MERS- PLpro of the selected primary hits. The ubiquitin-like peptide substrate was used in both (C) and (D) taking GRL0617 as a control. (E) Dose-response relationships of selected antiviral compounds, depicting both infectivity (colored), cytotoxicity (black), and EC_50_ values

Coronaviruses are taxonomically divided into multiple genogroups (alpha, beta, gamma, and delta), but human-infecting coronaviruses are found only among the alphacoronaviruses and betacoronaviruses thus far. The SARS-CoV-2-PLpro (SARS2-PLpro) shares 51.1% amino acid sequence similarity with that of MERS-CoV (MERS-PLpro) (Fig. 1A). Due to this moderate level of homology and medical importance, they were prioritized in this pairwise study for the development of broad-spectrum coronavirus PLpro inhibitors. SARS-CoV-2 and MERS-CoV each encodes only one PLpro domain within nsp3, which is an ortholog to the PLP2 domain from other coronaviruses encoding two PLpro domains. Although coronavirus PLpro’s catalyze the same chemical reaction, i.e., hydrolysis of peptide and isopeptide bonds, these closely related orthologs can differ significantly in terms of substrate recognition, enzymatic activity and inhibition by small-molecule compounds. For example, SARS2-PLpro preferentially cleaves ISG15 from substrates over ubiquitin chains, whereas SARS1-PLpro targets ubiquitin chains more than ISG15 (Shin et al. 2020). SARS1-PLpro shows more robust catalytic activity than MERS-PLpro toward most substrates and exhibiting a unique bivalent recognition mechanism toward polyubiquitin substrates. Both enzymes are capable of recognizing and hydrolyzing fluorophores from the C-termini of RLRGG peptide, Ub, and ISG15 substrates, yet the kinetic parameters associated with these reactions are different (Baez-Santos et al. 2014). Despite that a number of SARS2-PLpro and SARS1-PLpro inhibitors have been reported, none of these reports have convincingly demonstrated the *in vivo* activity of the identified drug compounds (Ratia et al. 2008; Baez-Santos et al. 2014; Baez-Santos et al. 2015; Osipiuk et al. 2021; Shan et al. 2021; Zhao et al. 2021). Moreover, the reported coronavirus PLpro inhibitors are virus-specific and cannot serve as a ready-to-use treatment option for future coronavirus epidemics. We hypothesized that broad-spectrum coronavirus PLpro inhibitors could be identified through screening of a large and structurally-diverse compound library. Herein, we demonstrate a broad-spectrum anti-coronavirus PLpro inhibitor, named F0213, with *in vivo* antiviral efficacy in lethal and non-lethal animal models. The identification of F0213 as a tool compound in our study may provide valuable information to optimize the rationale design of next-generation coronavirus PLpro inhibitors.

## Results

### Identification of PL-pro inhibitors by high-throughput screening

To identify potential PLpro inhibitors, a fluorescence-based high-throughput screen targeting the SARS2-PLpro cleavage activity was performed. In the interest of developing noncovalent inhibitors, DTT (5 mmol/L) was incorporated into all assays. A primary screen of 50,080 diverse and drug-like compounds was performed in 384-well plates. Only a small number of compounds, i.e., 54 (0.1%), were found to have more than 40% inhibitory activity toward PLpro (Fig. 1B). These primary hits were subjected to a series of confirmatory and secondary assays to exclude the interference from AMC fluorescence, followed by dose-dependent validations against both SARS2-PLpro and MERS2-PLpro. A class of 5-oxo-1-thioxo-4,5-dihydro[1,3]thiazolo[3,4-a]quinazoline-3-carboxamide molecules, designated F0213, F0326 and F0393, exhibited an IC_50_ of 7.4 µmol/L, 8.2 µmol/L and 15.8 µmol/L against SARS2-PLpro, respectively (Fig. 1C). As expected, GRL0617 (Ratia et al. 2008) potently inhibited SARS2-PLpro (IC_50_ = 1.1 µmol/L) but was ineffective against the MERS-PLpro activity (Fig. 1D). In contrast, the 3 hit compounds suppressed MERS-PLpro with IC_50_ ranging from about 10–20 µmol/L. To prioritize the compounds, we performed antiviral assessment with live SARS-CoV-2. Apparently, F0213 (EC_50_ = 4.5 µmol/L) and F0326 (EC_50_ = 7.6 µmol/L) harbor anti-SARS-CoV-2 activity at non-toxic concentrations, whereas F0393 showed considerable cytotoxicity when >10 µmol/L was utilized (Fig. 1E). Considering the lowest EC_50_ of F0213 against both SARS-CoV-2 and MERS-CoV (Figs. 1E and S1A), F0213 was chosen for subsequent characterization.

### F0213 is a broad-spectrum anti-coronavirus inhibitor

**Figure 2:**
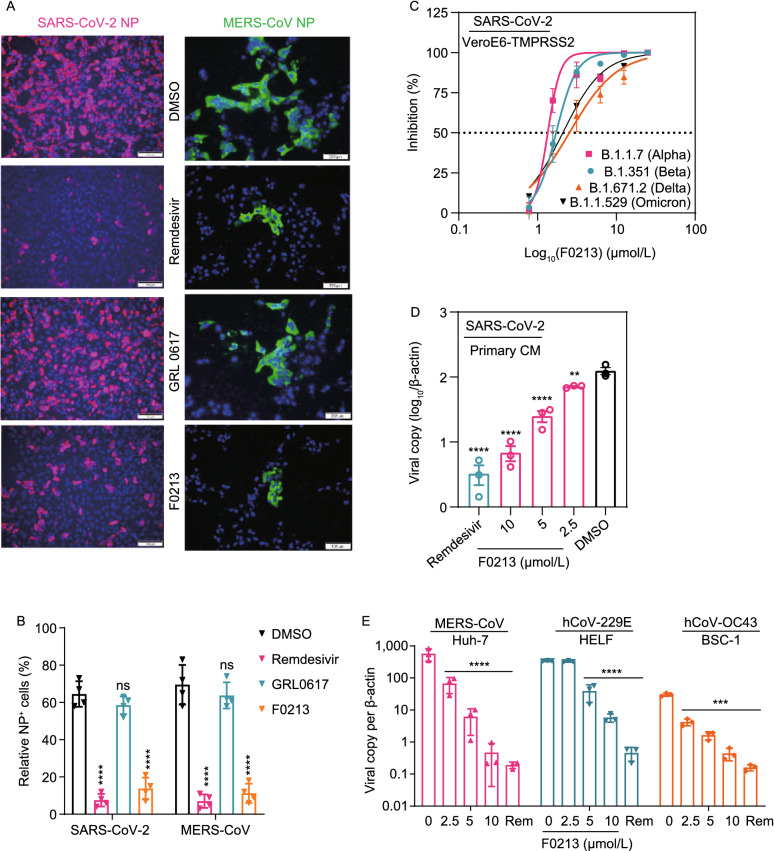
**F0213 inhibits a broad-spectrum of human-pathogenic CoVs replication in human cellular models**. (A) Immunofluorescence staining of SARS-CoV-2 NP antigen (Magenta) and MERS-CoV-NP antigen (green), and Vero cell nucleus (blue). Cells (0.1 MOI) were treated by DMSO (0.1%), Rmedesivir (10 µmol/L), GRL0617 (20 µmol/L), or F0213 (10 µmol/L) for 24 h, respectively. Shown are representative images selected from a pool images captured in two independent experiments. (B) Quantification of NP antigen signal using one-way ANOVA when compared with the DMSO group of either SARS-CoV-2 or MERS-CoV infection. ****P < 0.0001 and ns indicates *P* > 0.05. (C) Dose-response analysis of F0213 against SARS-CoV-2 variants of concern (Alpha, Beta, Delta and Omicron) in VeroE6-TMPRSS2 cells. EC_50_ was achieved by plaque reduction assays. (D) F0213 inhibited SARS-CoV-2 (0.1 MOI) replication in human primary CMs. Cell lysates were collected for viral load determination. Data represent mean ± SD for *n* = 3 biological replicates. (E) Antiviral activity of F0213 against MERS-CoV (0.01 MOI, 48 hpi), HCoV-229E (0.001 MOI, 72 hpi), and HCoV-OC43 (0.001 MOI, 72 hpi) in cell lines as indicated. Viral load in the cell lysate was quantified by RT-qPCR assays. Data represent mean ± SD for *n* = 3 biological replicates. One-way AVONA for statistical analysis were compared with the DMSO group (0 µmol/L), ****P < 0.0001, ***P < 0.001, **P < 0.01 and *P < 0.05

The antiviral potency of F0213 was also evidenced by the immunofluorescence staining of SARS2-NP or MERS-NP antigen that significant inhibition of virus replication was visualized after either remdesivir or F0213 treatment, while marginal virus suppression was observed when GRL0617 (20 µmol/L) was utilized in SARS-CoV-2-infected or MERS-CoV-infected Vero cells (Fig. 2A and 2B). To examine the antiviral efficacy of F0213 against SARS-CoV-2 variants of concern (VOC), plaque reduction assays were performed utilizing the emerging B.1.1.7 (Alpha), B.1.351 (Beta), B.1.617.2 (Delta) and B.1.1.529 (Omicron) variants on VeroE6-TMPRSS2 cells. Addition of F0213 suppressed replication of each VOC in a dose-dependent manner, exhibiting an EC_50_ ranging from 2.2–4.8 µmol/L (Fig. 2C). We then characterized the antiviral activity of F0213 in cardiomyocytes derived human embryonic stem cells (CM), which are more physiologically relevant and robustly support SARS-CoV-2 replication (Sharma et al. 2020). F0213 treatment reduced the SARS-CoV-2 viral yields to more than 1 log_10_, indicating its potential utility for amelioration of SARS-CoV-2 induced cardiac pathogenesis (Fig. 2D). To explore whether F0213 confers cross-protection against other epidemic and seasonal coronaviruses, we performed viral-load reduction assays for MERS-CoV, hCoV-229E and hCoV-OC43 in corresponding cell lines that support virus replication. Viral yields in cell culture supernatants were decreased by about 3 log10 in Huh-7 cells infected with MERS-CoV, by about 2 log10 in human embryonic lung fibroblasts infected with hCoV-229E and by around 1.5 log10 in monkey BSC-1 cells infected with hCoV-OC43 (Fig. 2E). Notably, F0213 showed negligible cytotoxicity in the matching cell lines as described above for broad-spectrum anti-coronavirus inhibitory evaluation (Fig. S1B). Overall, F0213 exhibited broad-spectrum anti-coronavirus efficacy, and antagonized SARS-CoV-2 replication in a human primary cell model.

### F0213 antagonizes PLpro-mediated innate suppression

PLpro has the function of stripping ubiquitin and ISG15 from host-cell proteins to aid coronaviruses in their evasion of the host innate immune responses (Baez-Santos et al. 2014; Shin et al. 2020). To explore the role of F0213 to rewire such process, cleaving activity against ubiquitin-AMC and ISG15-AMC substrates were titrated with different concentrations of F0213. Generally, MERS-PLpro showed higher sensitivity than that SARS-CoV-2 upon the drug treatment (Fig. S2A), corroborating that F0213 exhibits a lower antiviral EC_50_ against MERS-CoV (1.54 µmol/L) than SARS-CoV-2 (4.55 µmol/L). Structural and functional studies have revealed that PLpro is homologous to human deubiquitinating enzymes (DUBs) thus cleaving ubiquitin and ubiquitin-like modifiers such as ISG15 (Baez-Santos et al. 2014; Shin et al. 2020). To examine the specificity of F0213 for PLpro over human DUBs, we probed the ability of human DUBs from cellular lysates to be modified by the active-site-directed probe HA-Ub-vinyl sulfone (VS) in the presence and absence of F0213. In principle, cellular DUBs become modified when treated with HA-Ub-VS thus can be visualized by Western blot analysis by using an anti-HA antibody. Apparently, treatment with a positive control inhibitor NEM diminished the modification of HA-Ub-VS on DUBs derived from Caco-2 cells, whereas no change was noted in the immunoblot pattern upon F0213 treatment (left panel, Fig. S2B). When PLpro was added to the lysate, it too underwent modification by the HA-Ub-VS, but unlike the cellular DUBs, its modification by the VS was almost completely eliminated in the presence of F0213 (right panel, Fig. S2B). The results suggest that F0213 is a specific SARS2-PLpro deubiquitinating inhibitor. Subsequently, we explored the specificity of F0213 against cellular proteases derived from human lung A549 cells, hepatic Huh7 and colorectal Caco-2 cells. Utilizing a fluorescent-casein substrate, a wide variety of cellular proteases including serine proteases (trypsin, chymotrypsin, thrombin, plasmin, elastase, subtilisin), cysteine proteases (papain, cathepsin B) and acid proteases (thermolysin, pepsin) were not affected by F0213 (Fig. S2C).

To ascertain the role of F0213 to antagonize the PLpro-mediated IFN suppression, luciferase reporter assays reflecting the transcriptional activation of IFN-β, IRF3 and NF-κB genes were performed. Both SARS-CoV-2 and MERS-CoV attenuated IFN response, while addition of F0213 rescued the genes expression in a dose-dependent manner (Fig. S3A). In an infectious scenario, F0213 treatment significantly enhanced the expression of IFN-responsive genes Interferon-stimulated gene 15 (ISG15) and protein kinase R (PKR) in SARS-CoV-2-infected Caco-2 cells (Fig. S3B). By contrast, transcription levels of ISG15 and PKR were reduced by F0213 in a MERS-CoV-infected Huh-7 cell model (Fig. S3C). The differential patterns might be explained by the previous reports that SARS-CoV-2 infection in animal models and in COVID-19 patients is correlated with low IFN type I and type III responses (Blanco-Melo et al. 2020), whereas MERS-CoV generally elevated IFN response after infection (Inandiklioglu and Akkoc 2020). F0213, predominantly as a virus-targeting inhibitor, thus reverse the CoVs-induced IFN responses. In the absence of CoVs infection, intriguingly, F0213 potentiated the cellular antiviral signaling in both Caco-2 and Huh-7 cells (Fig. S3B and S3C). These findings suggest that F0213 may serve as a PLpro inhibitor to reverse the virus-induced immune dysregulation.

### F0213 exhibits distinct binding modes against SARS2-PLpro and MERS-PLpro

**Figure 3:**
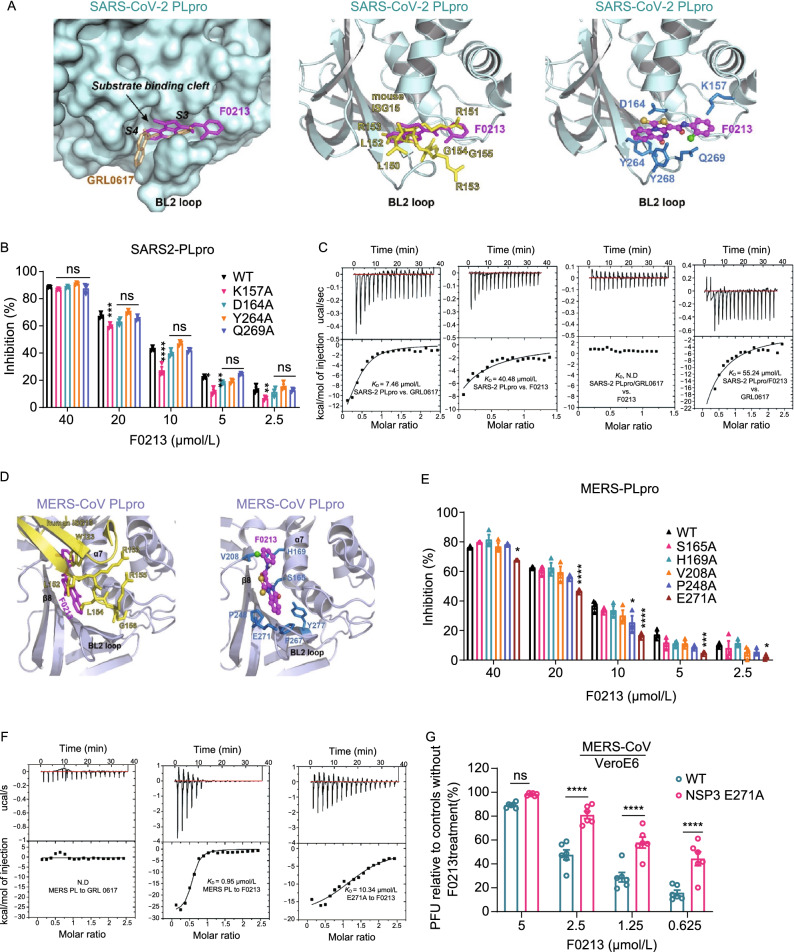
**Interaction between F0213 and SARS2-PLpro or MERS-PLpro**. (A) Docking F0213 to SARS2-PLpro. Left, molecular surface of SARS2-PLpro (colored cyan) with GRL0617 (colored gold, PDB: 7JRN) and F0213 (colored magenta, docking model) shown in stick model. The substrate binding cleft and the BL2 loop near active site is indicated. Middle, ribbon model of SARS2-PLpro with bound mouse ISG15 (colored yellow, PDB: 6YVA). The C-terminus of mISG15 is shown with the stick model. The predicted binding mode of F0213 (magenta) is shown with stick model. Right, detailed interaction between F0213 and SARS2-PLpro; residues were predicted to interact with the inhibitor are shown with the stick models (blue). (B) *In vitro* inhibition of WT and mutant SARS2-PLpro by F0213. Fixed concentration of PLpro (0.1 µmol/L) and 5 µmol/L of RLRGG-AMC substrate were incubated with serial-diluted F0213. Two-way ANOVA when compared with the WT % inhibition of in each F0213 concentration. (C) Isothermal titration calorimetry (ITC) experiments for the binding between SARS2-PLpro and inhibitors as indicated. Disassociation constant *K*_D_ is indicated; N.D. stands for non-detectable. (D) Docking F0213 to MERS- PLpro. Left, ribbon model of MERS-PLpro (colored light blue) bound by human ISG15 (colored yellow, PDB: 6BI8) is overlaid with the predicted binding mode of F0213 (colored magenta). The BL2 loop is indicated. Right, detailed interaction between F0213 and MERS-CoV PLpro; residues that were predicted to interact with the inhibitor are shown with the stick models and colored blue. (E) *In vitro* inhibition of WT and mutant MERS-PLpro by F0213. Two-way ANOVA when compared with the WT % inhibition of in each F0213 concentration. (F) ITC experiments for the binding between MERS-PLpro (WT or mutant) and inhibitors as indicated. Disassociation constant *K*_D_ is indicated; N.D. stands for non-detectable. (G) Recombinant virus carrying the E271A substitution in MERS-CoV NSP3 confers resistance to F0213. VeroE6 cells were infected with wild-type or mutant MERS-CoV generated by reverse genetics. Antiviral activities were determined by plaque assay detecting the live virus particle in the supernatant. Results are shown as the ratio between F0123-treated and vehicle treated groups that were infected by the same virus. Two-way ANOVA. For all statistical analysis, ****P < 0.0001, ***P < 0.001, **P < 0.01,*P < 0.05

To reveal the structural mechanism underlying the action of F0213, we sought to co-crystallize the compound with SARS2-PLpro. However, we were unable to obtain the structure complex. The difficulty was partially due to relatively low binding affinity of F0213 against SARS2-PLpro (*K*_d_ = 40.48 µmol/L), which is around 5-fold less than that of GRL0617 (*K*_d_ = 7.46 µmol/L) (Fig. 3C). Therefore, we used software Autodock Vina for docking the compound to the structures of SARS2-PLpro and MERS-PLpro protease, respectively. With a search space covering the substrate binding pockets of SARS2-PLpro (PDB ID: 7JRN), we identified 20 binding modes of F0213, the best of which had a predicted binding affinity −8.0 kcal/mol. This model predicted that the interaction between F0213 and SARS-CoV-2 PLpro involves five key residues: K157, D164, Y264, Y268 and Q269. In particular, F0213 was placed inside a narrow substrate binding cleft near the active site of the protease (Fig. 3A). The thiazolo-quinazoline rings of F0213 occupied the S3-S4 pockets of SARS-CoV-2 where it attached tightly to the blocking loop 2 (BL2 loop), possibly via π-π stacking with the side chain of Y268 on the tip of the BL2 loop. Structure superimposition revealed that the narrow cleft is also occupied by the benzenamine moiety of GRL0617 or the leucine (P4) of the LRGG motif of ISG15 (PDB ID: 6YVA). On the other hands, the chlorophenyl moiety of F0213 overlays with the position of R151 of the bound ISG15, an important residue for ISG15 mediated antiviral immune response (Giannakopoulos et al. 2009). To validate this prediction, we employed site-mutagenesis to construct individual SARS2-PLpro mutant (Fig. S4A). All mutant proteins were enzymatic active except Y268A. To examine the inhibitory activity of F0213 against each mutant, protease cleavage assay using RLRGG-AMC substrate was conducted. Only K157A substitution diminished the inhibitory potency of F0213 in the concentrations of 20, 10, 5, and 2.5 µmol/L, while the others did not (*P* < 0.01, Fig. 3B). Collectively, the results suggest that K157 of SARS2-PLpro is a key amino acid residue mediating SARS2-PLpro/F0213 interaction.

As hinted from the docking analysis, we performed competitive binding experiments to validate whether the binding site of F0213 overlaps with that of GRL0617. When GRL0617 was pre-incubated with SARS2-PLpro, heat exchanges induced by the addition of F0213 were undetectable by isothermal titration calorimetry, indicating that GRL0617 shields the binding surface of F0213 on SARS2-PLpro (Fig. 3C). When F0213-SARS2-PLpro complex was formed before GRL0617 addition, the resulted binding affinity (*K*_D_ = 55.24 μmol/L) dropped about 8-fold when compared with SARS2-PLpro alone (*K*_D_ = 7.46 μmol/L, Fig. 3C). The results imply that F0213, like GRL0617, may act as a competitive inhibitor against SARS2-PLpro, which induces a loop closure that shuts down catalysis at the SARS2-PLpro active site.

Using a similar approach, we docked F0213 to a crystal structure of MERS-PLpro (PDB ID: 4RNA) and identified 20 binding modes of the compound. The best binding mode had a predicted binding affinity −7.9 kcal/mol and involved seven key residues of MERS-CoV PLpro: S165, H169, V208, P248, F267, E271 and Y277. The predicted binding mode of MERS-PLpro/F0213 differed significantly from that with SARS2-PLpro. Instead of attaching to the BL2 loop, F0213 is inserted to a shallow cleft formed between α7 and β8 of MERS-PLpro (Fig. 3D). Superimposing our model with human ISG15-MERS-PLpro complex (PDB ID: 6BI8) reveal that the chlorophenyl moiety of the modelled F0213 overlays with residue W123 of MERS-PLpro, which is an important residue mediating hydrophobic interaction between MERS-PLpro and ISG15 (Clasman et al. 2020). Biochemical validation showed that either F267A or Y277A substitution diminish the MERS-PLpro enzyme activity, whereas E271A substitution compromised the inhibitory efficacy of F0213 against MERS-PLpro (Figs. 3E and S4B). As expected, GRL0617 exhibited no binding affinity against MERS-PLpro (Fig. 3F). Notably, the kD between MERS-PLpro_WT and F0213 drops from 0.95 µmol/L to that of 10.34 µmol/L (>10 fold) when the E271A substitution was introduced (Fig. 3F). The result suggests that E271 amino acid residue is critical to mediate MERS-PLpro and F0213 interaction.

To explore if the identified K157A and E271A mutations render resistance to F0213, we sought to generate recombinant SARS-CoV-2-NSP3-K157A and MERS-CoV-NSP3-E271A viruses, respectively. We successfully rescued the MERS-CoV-NSP3-E271A, but not the recombinant SARS-CoV-2 containing K157A mutation from three independent experiments. As shown in Fig. 3G, F0213 inhibited the mutant MERS-CoV significantly less than the WT MERS-CoV (*P* < 0.0001) at different concentrations. The result again verified that 271E is crucial for F0213 to exert MERS-PLpro inhibition.

## F0213 improve lung pathogenesis in a SARS-CoV-2 hamster model

**Figure 4:**
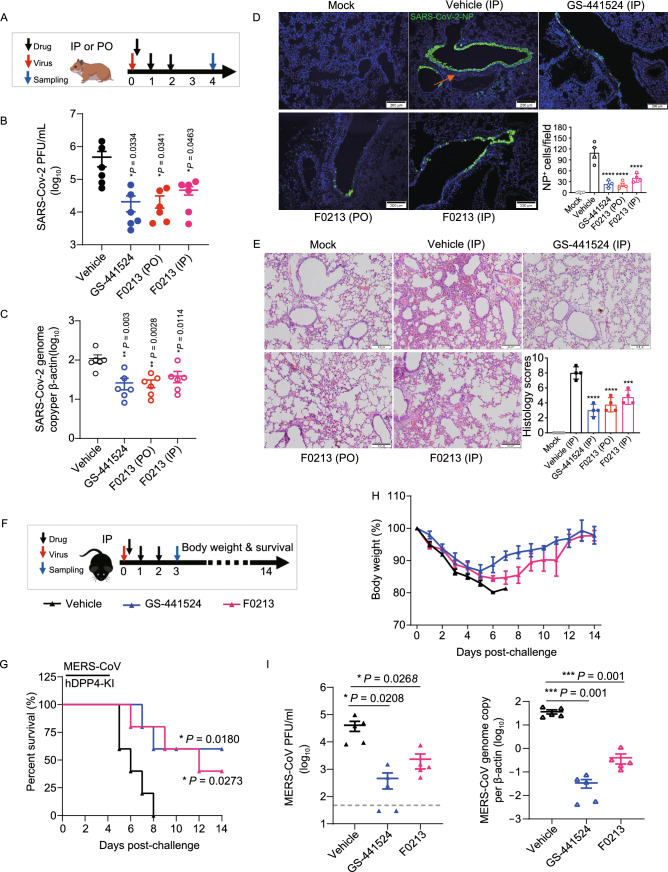
**Antiviral efficacy of F0213 in animal models**. (A–E) F0213 improves lung pathogenesis in a SARS-CoV-2 hamster model. (A) Therapeutic treatment used oral (PO) or intraperitoneal (IP) administration of F0213 (5 mg/kg), given at 6 hpi, 24 hpi and 48 hpi after virus challenge at day 0. Lung tissue samples were collected at 4dpi. GS-441524 (40 mg/kg) was included as a control via IP route. Vehicle contains 2% DMSO in 12% SBE-β-CD and by IP injection. (B and C) Viral yields in hamster lungs were determined by plaque assay (B) and RT-qPCR assay (C), respectively. (D) Representative images of infected cells by immunofluorescence staining in lung. SARS-CoV-2 N expression (green) is shown in diffuse alveolar areas (red arrow) of vehicle group, which is absent in other drug-treated groups. N-positive cells per 50× field per hamster lung section. (E) Representative images of H&E-stained lung tissue section from hamsters treated as indicated, followed by semi-quantitation of histology scores given to each lung tissue by grading the severity of damage in bronchioles, alveoli and blood vessels and accumulating the total scores. Scale bars, 200 μm. One-way ANOVA followed by Dunnett’s post test and compared with vehicle control. ****P < 0.0001, ***P < 0.001. (F–I) F0213 protects mouse from lethal MERS-CoV challenge. (F) Therapeutic treatment used intraperitoneal (IP) administration of F0213 (20 mg/kg), given at 6 hpi, 24 hpi and 48 hpi after virus challenge at day 0. Lung tissue samples were collected at 3 dpi. GS-441524 (40 mg/kg) was included as a control via IP route. Vehicle contains 2% DMSO in 12% SBE-β-CD and by IP injection. (G) Survival and clinical disease were monitored for 14 days or until death. *P < 0.05 by log-rank (Mantel-Cox) tests. (H) Daily body weights of surviving mice. (I) Lung tissues were collected for detection of viral titers at 3 dpi. A value of 30 PFU/mL was assigned for any titer below the 50 PFU/mL detection limit (the dotted line). One-way ANOVA when compared with the vehicle group. *P < 0.05 and **P < 0.01

Next, we utilized our established golden Syrian hamster model to evaluate the anti-SARS-CoV-2 activity of F0213 *in vivo* (Chan et al. 2020). Therapeutic regimen using either oral (PO) or intraperitoneal (IP) administration of F0213 (5 mg/kg) was applied, with the first dose given at 6 hpi and once daily for consecutive 3 days (Fig. 4A). On 4 dpi when viral loads peaked with substantial histopathological changes, F0213 decreased the infectious viral plaque-forming units (PFU) in lung tissues by about 1–2 log_10_, which was comparable with the effect of IP GS-441524 (Fig. 4B). Consistently, a comparable degree of suppression of SARS-CoV-2 genome copies (normalized by β-actin) in the lungs were observed in F0213- or GS-441524 -treated hamsters (Fig. 4C). Immunofluorescence staining suggested abundant SARS-CoV-2 N expression (green) as shown in diffuse alveolar areas and in the focal bronchiolar epithelial cells of the vehicle-treated hamster lungs, whereas both GS-441524- and F021-treated groups exhibited reduced N expression (Fig. 4D). The severity of lung damage was also examined by performing hematoxylin and eosin (H&E) staining. Expectedly, vehicle-treated group developed alveolar consolidation and inflammatory infiltration (Fig. 4E). In contrast, F0213- or GS-441524-treated hamsters exhibited improved morphology and milder inflammatory infiltration with significantly reduced lung histology scores (Fig. 4E). Overall, our findings demonstrated that F0213 conferred protection against SARS-CoV-2 challenge in the golden Syrian hamster model by reducing virus replication and associated inflammatory damage.

### F0213 protects mouse from lethal MERS-CoV challenge

To determine the anti-MERS-CoV potency of F0213 *in vivo*, we employed a lethal human dipeptidyl peptidase-4 (DPP4) knock-in mouse model as previously described (Li et al. 2017). We challenged mice with 2000 PFU of mouse-adapted MERS-CoV, followed by IP delivery of F0213 (20 mg/kg) or GS-441524 (40 mg/kg) at 6 hpi, 24 hpi and 48 hpi, respectively (Fig. 4F). All vehicle–treated mice died on or before 8 dpi, whereas F0213 treatment resulted in higher survival rate (40% vs. 0%, *P* = 0.0273) (Fig. 4G). Generally, less body weight loss was recorded in both F0213- and GS-441524-treated groups, with the mice having started to rebound at 8 dpi and 6 dpi, respectively (Fig. 4H). The improved clinical disease was also evidenced by >1-log_10_ reduction of both live virus PFU and viral genome copies (normalized by β-actin) in mouse lungs on 3 dpi (Fig. 4I). Taken together, F0213 effectively protected animals challenged with SARS-CoV-2 or MERS-CoV by reducing virus replication.

## Discussion

Targeting the three distinct substrates of PLpro, namely the viral polyprotein, degradative Lys48-polyubiquitin and antiviral ISG15 signals, we demonstrate here the coronavirus PLpro to be a feasible broad-spectrum target for various human-pathogenic coronaviruses. Despite the considerable efforts lasting from SARS to MERS, and now COVID-19, none of the reported coronavirus PLpro inhibitors has been shown to be active in animal models. Pharmacokinetic characterization of F0123 is warranted to achieve enhanced metabolic stability *in vivo*, the major obstacle hindering anti-PLpro drug development (Ratia et al. 2008; Baez-Santos et al. 2014; Baez-Santos et al. 2015; Osipiuk et al. 2021; Shan et al. 2021; Zhao et al. 2021). Compared with GRL0617 and its analogue, a “model” compound that have been extensively analyzed in our and others’ studies on SARS1 or SARS2-PLpro (Ratia et al. 2008; Shin et al. 2020; Fu et al. 2021; Gao et al. 2021), the versatile binding modes of F0213 may represent as a new generation of ‘tool’ compound to facilitate rational design of pan-coronavirus PLpro inhibitors.

The SARS2-PLpro enzyme has virtually identical substrate specificity as its homologs from SARS-CoV (both lineage B betacoronaviruses), but differs significantly from that of MERS-PLpro (lineage C betacoronavirus) (Baez-Santos et al. 2014). The overall MERS-PLpro structure is similar to that of SARS2-PLpro and SARS1-PLpro. Both PLpro enzymes contain two blocking loops named BL1 and BL2 that could be structurally important. However, the flexible BL2 loop of MERS-PLpro differs from that of SARS2-PLpro and SARS1-PLpro, which raised the possibility of different roles in inhibitor binding. This may explain the observation that all of the previously tested SARS-PLpro lead inhibitors were ineffective against MERS-PLpro. For example, GRL0617 resides in a pocket in the palm region of PLpro, which is apart from the catalytical triad including C111, H272, and D286 (Fu et al. 2021). The inactivity of GRL0617 against MERS-CoV is due to the lack of critical Y268 of SARS2-PLpro encircling GRL0617 because the flexible BL2 loop is positioned much further from its active site than that of SARS2-PLpro when it is unbound. Similarly, F0213 does not bind to the catalytic site of the SARS2-PLpro enzyme but to the BL2 loop, blocking the entrance of the active site (Fig. 3). Instead of attaching to the BL2 loop, however, F0213 is inserted to a shallow cleft formed between α7 and β8 of MERS-PLpro, probably inducing conformational changes. These results suggest that inhibitor recognition specificity of MERS-PLpro may differ from that of SARS2-PLpro and SARS1-PLpro even though the overall structures of the whole protein and the catalytic sites are very similar. The most probable contributing factor for inhibitor selectivity of these two PLpro enzymes could be attributed to the structural differences of the BL2 loop.

Our study had limitations. Despite numerous efforts applying both co-crystallization and crystal socking strategies, crystallization of PLpro-F0213 complex was unsuccessful thus far. The failure in crystallization of PLpro-F0213 complex was probably due to the low binding affinity of F0213 (*K*_d_ = 40.48 μmol/L) when compared with that of GRL0617 (*K*_d_ = 7.46 μmol/L). Structural optimization of F0213 with enhanced binding and inhibitory activity against PLpro should be considered in future studies. It would also be important to fully document the pharmacokinetics of F0213 and/or its analogues in different animal models to facilitate the rational design of therapeutic regimes.

In summary, F0213 is the first broad-spectrum anti-coronavirus PLpro inhibitor that is orally available, which offers a potential at-home treatment option for COVID-19 and future coronavirus infections. This broad-spectrum anti-coronavirus inhibitor warrants further developments to combat the current COVID-19 pandemic and future coronavirus outbreaks.

## MATERIALS AND METHODS

### Viruses and cells

SARS-CoV-1 and SARS-CoV-2 (lineage B βCoV), MERS-CoV (lineage C βCoV), hCoV-OC43 (lineage A βCoV), and hCoV-229E (αCoV) were included in this study to represent the subgroups of CoVs that cause human infections. The WT SARS-CoV-2 HKU-001a (GenBank accession number MT230904), B.1.1.7/Alpha (GISAID: EPI_ISL_1273444), B.1.351/Beta (GISAID: EPI_ISL_2423556), B.1.617.2/Delta (GISAID: EPI_ISL_3221329), and B.1.1.529/Omicron (GISAID accession number EPI_ISL_7138045) strains were isolated from respiratory tract specimens of laboratory-confirmed COVID-19 patients in Hong Kong (Yuan et al. 2021). MERS-CoV (EMC/2012) was kindly provided by Ron Fouchier (Erasmus Medical Center, the Netherlands). Archived clinical strains of SARS-CoV-1 (GZ50 strains, GenBank accession number: AY304495), HCoV-OC43, and HCoV-229E were obtained from the Department of Microbiology, The University of Hong Kong (HKU). All the cell lines used in this study, except for HEL (human embryonic lung fibroblasts; in-house development), were obtained from American Type Culture Collection. Cardiomyocytes derived human embryonic stem cells (CM) were prepared as we previously described (Yuan et al. 2021). The inhibitory activity of F0213 against 229E replication were tested in HEL cells and OC43 in BSC-1 cells.

### Chemical reagents

The Smart^TM^ chemical library was purchased from Chemdiv (San Diego, USA), which contains 50,080 small-molecule compounds with enhanced diversity and drug-like properties. Remdesivir and its prodrug GS-441524, as well as GRL0617 were purchased from MedChemExpress (NJ, USA). Arg-Leu-Arg-Gly-Gly-AMC (RLRGG-AMC) was purchased from Bachem Bioscience (Bubendorf, Switzerland). ISG15-AMC, ubiquitin-AMC, and HA-Ub-VS were purchased from R&D systems (Minneapolis, USA). Cellular protease assay kit (Cat# 539125) was purchased from Sigma-Aldrich. CellTiter-Glo cell viability assay kit was purchased from Promega. Other chemicals were purchased from Sigma-Aldrich unless specified.

### PLpro phylogenetic analysis

Nsp3 PLpro protein sequence alignments and phylogenetic trees were generated using Clustal Omega (Sievers and Higgins 2018), Mega X (Kumar et al. 2018) and visualized using ggtree (Yu et al. 2018). Protein similarity and BLOcks SUbstitution Matrix 62 (BLOSUM62) score were calculated using BLOSUM62 matrix (Gu et al. 2016). The accession numbers used were as follows: PDCoV (KR265858), hCoV-229E (JX503060), hCoV-HKU1 (DQ415904), hCoV-NL63 (JX504050), hCOV-OC43 (AY903460), HKU5-1 (NC_009020), MERS-CoV (JX869059), HKU9-4 (EF065516), HKU3-1 (DQ022305), SHC014 (KC881005), WIV1 (KF367457), SARS-CoV (AY278741) and SARS-CoV-2 (NC_045512).

### Viral load reduction assay

Viral load reduction assay was performed by quantitative reverse transcription-polymerase chain reaction (qRT-PCR) as we described previously with slight modifications (Yuan et al. 2020). Briefly, RNA was extracted from culture supernatants of the CoV-infected cell lines as mentioned above using the MiniBEST Viral RNA/DNA Extraction Kit (Takara Bio Inc., Kusatsu, Shiga Prefecture, Japan). Reverse transcription was performed with the Transcriptor First Strand cDNA Synthesis Kit (Roche, Basel, Switzerland) with oligo-dT primers. To determine the virus genome copies, qPCR was performed using the LightCycler 480 SYBR Green I Master Mix (Roche) with specific primers. hCoV-229E_Forward: CTACAGATAGAAAAGTTGCTTT; hCoV-229E_Reverse: GGTCGTTTAGTTGAGAAAAGT; hCoV-OC43_ Forward: AAACGTGCGTGCATC; hCoV-OC43_ Reverse: AGATTACAAAAAGATCTAACAAGA; hCoV-NL63_ Forward: GGAGATAGAGAATTTTCTTATTTAGA; hCoV-NL63_ Reverse: GGTTTCGTTTAGTTGAGAAG. The virus genome copies in supernatant samples were quantified with a standard.

### Plaque reduction assay

Plaque reduction assay was performed in 24-well tissue culture plates as we described previously with slight modifications (Yuan et al. 2020). Briefly, Vero cells were seeded at 1 × 10^5^ cells/well in MEM (Invitrogen, Carlsbad, CA, USA) with 10% FBS on the day before the assay was carried out. After 16–24 h of incubation, 70–100 plaque-forming units (PFU) of SARS-CoV-2 or MERS-CoV was added to the cell monolayer with or without the addition of drug compounds and the plates further incubated for 2 h at 37 °C in 5% CO_2_ before removal of unbound viral particles by aspiration of the media and washing once with MEM. The cell monolayers were then overlaid with media containing 1% low melting agarose (Cambrex, East Rutherford, NJ, USA) in MEM and appropriate concentrations of the drug compounds and incubated as above for 72 h. Next, the wells were fixed with 10% formaldehyde overnight. After removal of the agarose plugs, the cell monolayers were stained with 0.7% crystal violet and the plaques counted. The percentage of plaque inhibition relative to the control (0.1% DMSO) plates was determined for each drug compound concentration.

### Expression and purification of PLpro

Recombinant SARS2-PLpro from the reference sequence Wuhan-Hu-1 (GenBank ID YP_009724390.1) (wild type) and each with point mutation of C111S, K157A, D164A, Y264A, Y268A or Q269A were codon-optimized and clone into pET28b+ expressed and purified in *E*. *coli* as we described previously with modifications (Yuan et al. 2016). MERS-PLpro (GenBank: JX869059.2) were expressed in the same way, including point mutation of S165A, H169A, V208A, P248A, F267A, E271A or Y277A, individually. The constructs were fused with an N-terminal 6× His tag for purification. The overexpression of the PLpro was induced by 0.1 mmol/L of IPTG at 16 °C for 16 h with agitation at 250 rpm. The concentration of purified PLpro was determined by using the Bradford Assay Kit (Bio-Rad) according to the manufacturer’s instructions. The purity of each recombinant PLpro mutant protein was verified by SDS-PAGE.

### Molecular docking

Crystal structures of SARS-CoV-2 and MERS-CoV papain-like protease (PLpro) used for molecular docking was retrieved from the Protein Data Bank (PDB) ID 7JRN and 4RNA21, respectively. Coordinate of the compound F0213 was downloaded from PubChem (Kim et al. 2021). Preparation of the macromolecules PLpro and the ligand F0213 for molecular docking was carried out using software AutoDockTools following standard protocols. The molecular docking was performed using program Autodock Vina with default parameters. Presentation of PLpro/F0213 interactions was generated using software PyMOL (The PyMOL Molecular Graphics System, Version 2.0 Schrödinger, LLC) with academy licenses.

### Protease cleavage assay

To explore the cleavage inhibition of PLpro against ISG15 and ubiquitin, ubiquitin–AMC or ISG15–AMC were used as substrate of PLpro and the release of AMC was measured by increase of fluorescence (excitation/emission, 360/487 nm) on a 384-well microplate reader (PHERAstar FSX, BMG Labtech). Twenty-five microlitres of solution containing different concentrations of F0213 (final concentrations range from 100 to 0 μmol/L) and 1 μmol/L of ubiquitin-AMC or ISG15-AMC was aliquoted into a 384 well plate with the reaction initiated by addition of 25 μL of PLpro (0.1 μmol/L) to the well. Initial velocities of AMC release was normalized against DMSO control. The IC_50_ value was calculated by the dose–response–inhibition function in Graphpad Prism with [inhibitor] vs. normalized response equation. GRL-0617 was used as a positive control throughout the experiments.

### Reporter gene assay

To analyze the induction of IFN-β induced genes in the presence/absence of PLpro, and with or without F0213 treatment, luciferase reporter assays were performed in 293T cells (Shin et al. 2020). In brief, an expression construct containing the luciferase ORF and the IFN-β promoter (IFN-β-luciferase) or NF-κb or IRF3 was co-transfected with either a pCAGEN control plasmid or the designated PLpro plasmid and Renilla plasmid. For all transfections, 100 ng of luciferase plasmid, 400 ng of PLpro or pCAGEN vector and 5ng Renilla plasmid were used in each well of a 24-well plate. Twenty-four hours after transfection, cells were treated with 500 ng poly(I:C) for 18 h or 50 ng/mL of TNF for 30 min. For the F0213 treatment group, serial diluted concentrations of F0213 was added to relative wells after 6 h poly(I:C) or 50 ng/mL TNF treatment. Luciferase expression was measured using the Luciferase Reporter Assay System (Promega). Fold change was calculated by taking vector treated with poly(I:C) as 1.

### Hamster experiment

Male and female Syrian hamster, aged 6–10 weeks old, were kept in biosafety level 3 housing and given access to standard pellet feed and water, as we previously established (Yuan et al. 2021). All experimental protocols were approved by the Animal Ethics Committee in the HKU (CULATR) and were performed according to the standard operating procedures of the biosafety level 3 animal facilities (reference code: CULATR 5370-20). Experimentally, each hamster was intranasally inoculated with 10^5^ PFU of SARS-CoV-2 in 100 µL PBS under intraperitoneal ketamine (200 mg/kg) and xylazine (10 mg/kg) anaesthesia. Six-hour post-virus-challenge, hamsters were intraperitoneally given either F0213 (5 mg/kg), or GS-441524 (40 mg/kg) or vehicle control (2% DMSO in 12% SBE-β-CD) for consecutive 4 days. Animals were monitored twice daily for clinical signs of disease. Six animals in each group were sacrificed at 4 dpi for virological and histolopathological analyses as we previously described (Gu et al. 2016). Viral yield in the lung tissue homogenates was detected by plaque assay and qRT-PCR method, respectively.

### Human DPP4-knockin (hDPP4-KI) mouse experiment

The hDPP4 exon 10–12 knockin mice were provided by Dr. Paul McCray (University of Iowa, IA, USA). All experimental protocols were approved by the CULATR and were performed according to the standard operating procedures of the biosafety level 3 animal facilities (reference code: CULATR 5193-19). Littermates of the same sex were randomly assigned to experimental groups. On the day of infection, the mice were intranasally inoculated with 2000 PFU mouse-adapted MERS-CoV (MERS-CoV_MA_) as we previously described (Chu et al. 2021). Infected mice received same regimen as that of hamster experiment except the increasing dose of F0213 (20 mg/kg). Body weight of each mouse was monitored on daily basis for 14 days or until death. Virological analyses were performed after mice were sacrificed on 3 dpi.

### Isothermal titration calorimetry (ITC)

The binding affinity between SARS2-PLpro and F0213 or GRL0617 was measured under 25 °C through isothermal titration calorimeter (MicroCal iTC200, Malvern Panalytical) as we previously described (Gao et al. 2018). Both the protein and inhibitor were dissolved in the buffer containing 20 mmol/L HEPES, pH = 7.4, 150 mmol/L NaCl and 0.8% DMSO. The protein concentration in syringe ranged from 0.25 to 0.5 mmol/L while in reaction cell ranged from 0.03 to 0.06 mmol/L. The competition experiment was performed using the same conditions, except that the SARS-PLpro was pre-incubated with F0213 or GRL0617 at molar ratio 1:1. All titration data was calculated and analyzed by using a single-site binding model for nonlinear curve fitting in MicroCal ITC-ORIGIN Analysis Software (Malvern Panalytical). ITC titration was repeated at least twice for each experiment.

### Generation of recombinant MERS-CoV with NSP3 E271A mutation

Reverse genetic to generate recombinant MERS-CoV was performed as we previously described (Wong et al. 2020). MERS-CoV (EMC/2012) molecular clone in pBeloBAC11 backbone was kindly provided by Professor Luis Enjuanes (Spanish National Center for Biotechnology, Madrid, Spain). The point mutation (E271A) was generated in pBeloBAC11-EMC-2012 BAC using the lambda Red-mediated homologous recombination, followed by transfection to BHK-21 cells. After six hours, the cells were trypsinized and co-cultured with Vero cells to generate recombinant MERS-CoV carrying the NSP3 E271A mutation.

## Author contributors

All authors have read and agreed to this version of the manuscript. All data have been included in this manuscript. All materials are available to share upon reasonable request. S. Y., X. G., K. T., M. H., L. W. and B. Q. designed and/or performed experiments. J-P. C., and C. L. generated critical reagents. S. Y., S. C., J. F-W. C, and K-Y. Y wrote the manuscript. K.T., Y.H., J. C., R. L., and Z. Q. provided the animal data. Z-W. Y., performed histopathological evaluation and infectious clone. H. C., D-Y. J and R. S. provided conceptual advice and troubleshooting. S. Y., S. C., and K-Y. Y oversaw the conception and supervised the study. S. Y., S. C., J. F-W. C, and K-Y. Y provided the grant support.

## Supplementary Information

Below is the link to the electronic supplementary material.

Supplementary file1 (PDF 382 kb)

## Abbreviations

CM: cardiomyocytes
PLpro: papain-like protease
DMV: double-membrane vesicles
DPP4: dipeptidyl peptidase-4
DUB: deubiquitination
H&E: hematoxylin and eosin
hCoV: human coronaviruses
HEL: human embryonic lung fibroblasts
IFN: interferon
IP: intraperitoneal
IRF3: interferon regulatory transcription factor 3
ISG15: interferon-stimulated gene 15
ITC: isothermal titration calorimetry
MERS-Cov: middle East respiratory syndrome coronavirus
MOI: multiplicity of infection
NF-κB: nuclear factor kappa-light-chain-enhancer of activated B cells
NP: nucleocapsid protein
PFU: plaque-forming units
PKR: protein kinase R
PO: oral
qRT-PCR: quantitative reverse transcription-polymerase chain reaction
SARS-CoV: severe acute respiratory syndrome coronavirus
UB: ubiquitin
VOC: variants of concern
