# Supplementary material for: Targeting papain-like protease for broad-spectrum coronavirus inhibition
Source: Protein Cell. 2022 Apr 6;13(12):940–53. doi: 10.1007/s13238-022-00909-3 (PMC8983325; doi:10.1007/s13238-022-00909-3)
Supplement: Supplementary file 1 — Supplementary file1 (PDF 382 kb) [file 13238_2022_909_MOESM1_ESM.pdf]

Supplementary Materials for

Targeting papain-like protease for broad-spectrum coronaviruses inhibition

Shuofeng Yuan[1,2†*], Xiaopan Gao[3†], Kaiming Tang[2†], Jian-Piao Cai[2†], Menglong Hu[4], Peng Luo[2], Lei Wen[2], Zi-Wei Ye[2], Cuiting Luo[2], Yaoqiang Huang[2], Jianli Cao[2], Ronghui Liang[2], Zhenzhi Qin[2], Bo Qin[3], Hin Chu[1,2], Ren Sun[4,5], Jasper Fuk-Woo Chan[1,2*], Sheng Cui[3*], Kwok-Yung Yuen[1,2*]

This PDF file includes:

Figs. S1 to S4

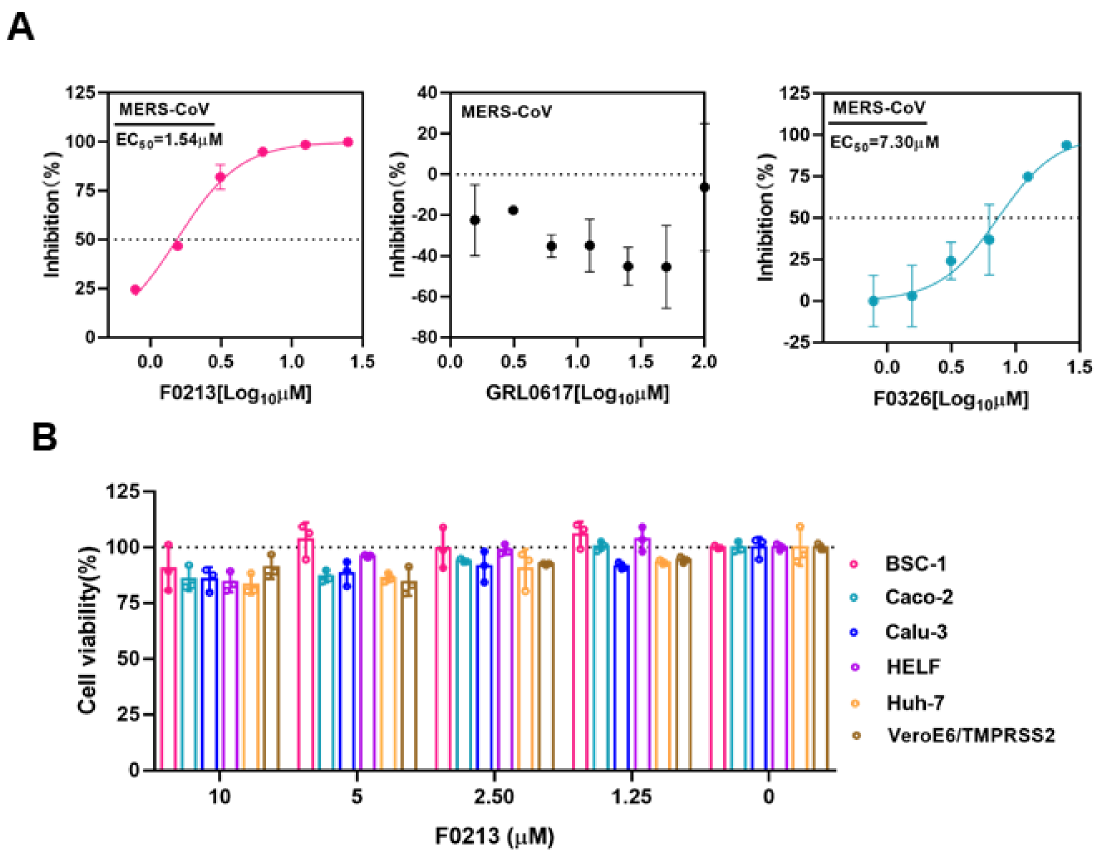

Figure S1. Anti-MERS-CoV activity and cytotoxicity measurement of F0213. (A) EC_{50} of F0213 or F0326 against MERS-CoV was plotted by plaque reduction assay. GRL0617 showed no inhibition against MERS-CoV. (B) Cell viability of F0213 in different cell lines were determined using CellTiter-Glo assays and in the absence of virus infection. The drug-incubation time in the cytotoxicity assay was consistent with that in the antiviral assays, e.g. at 24h post-treatment for Huh-7 cells; at 48h post-treatment for VeroE6/TMPRSS2 cells, Calu-3 and Caco-2 cells; and at 72h post-treatment for BSC-1 cells and human embryonic lung fibroblasts (HELF), respectively. Data represent mean ± SD for n=3 biological replicates. The experiment was repeated twice for confirmation.

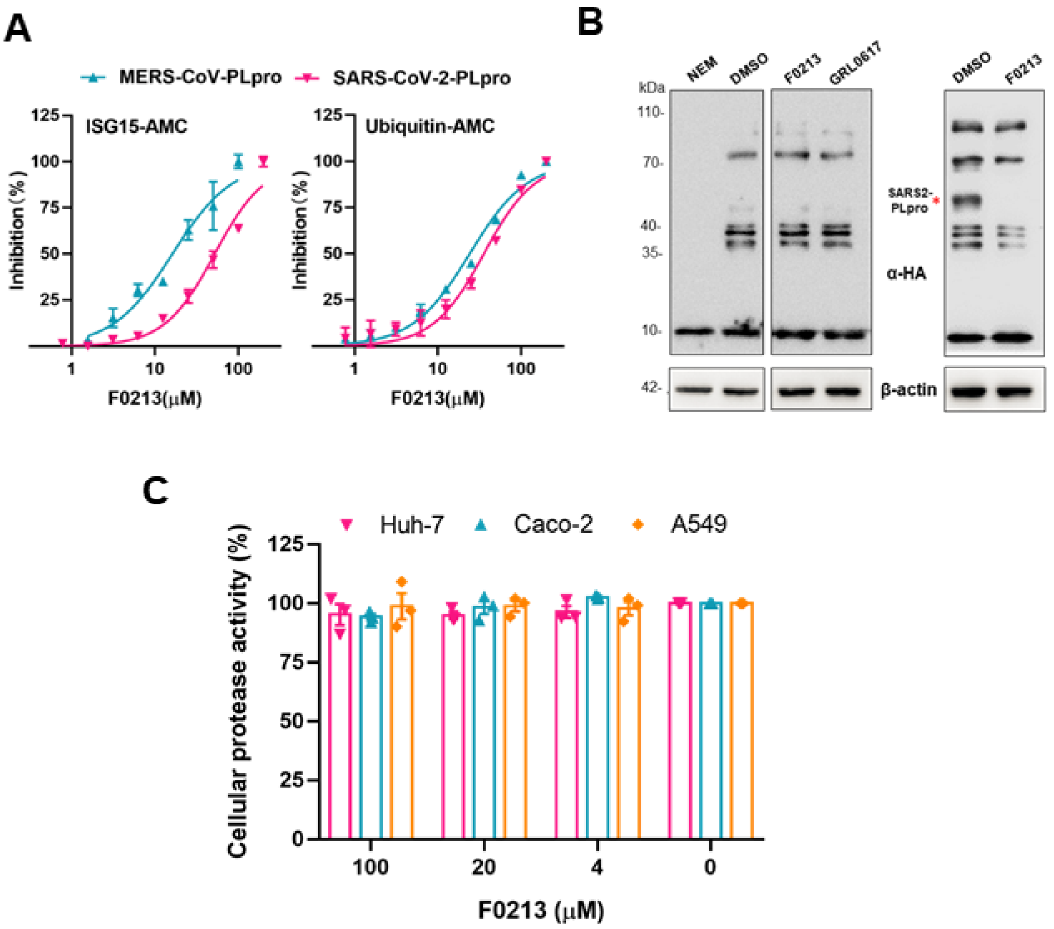

Figure S2. Specificity analysis of F0213. (A) F0213 inhibited cleavage of ISG15–AMC and Uniquitin-AMC that mediated by MERS-CoV PLpro and SARS-CoV-2 PLpro. Data are mean ± SD, n = 3 independent experiments. (B) Specificity of F0213 for PLpro over human DUBs. Left panel: An anti-HA Western blot of lysed human Caco-2 cells treated with HA-Ub-VS in the presence of N-ethyl-maleimide (NEM, positive control inhibitor) or GRL0617. Right panel: 0.1 μg of SARS2-PLpro was added to Caco-2 cell lysate before covalent modification by HA-Ub-VS, showing that F0213 eliminated PLpro-based modification. (C) Determination of the cellular protease activity in the presence of F0213 (100, 20, 4 and 0 μM). Lysates of human liver Huh-7 cells, human colon Caco-2 cells and human lung A549 cells were incubated with a fluorescent-casein substrate before reading, detecting a wide variety of proteases including serine proteases, cysteine proteases and acid proteases. Results were normalized with the readout of 0.1% DMSO (i.e. 0 μM) group. Data represent mean ± SD for n=3 biological replicates. The experiment was repeated twice for confirmation.

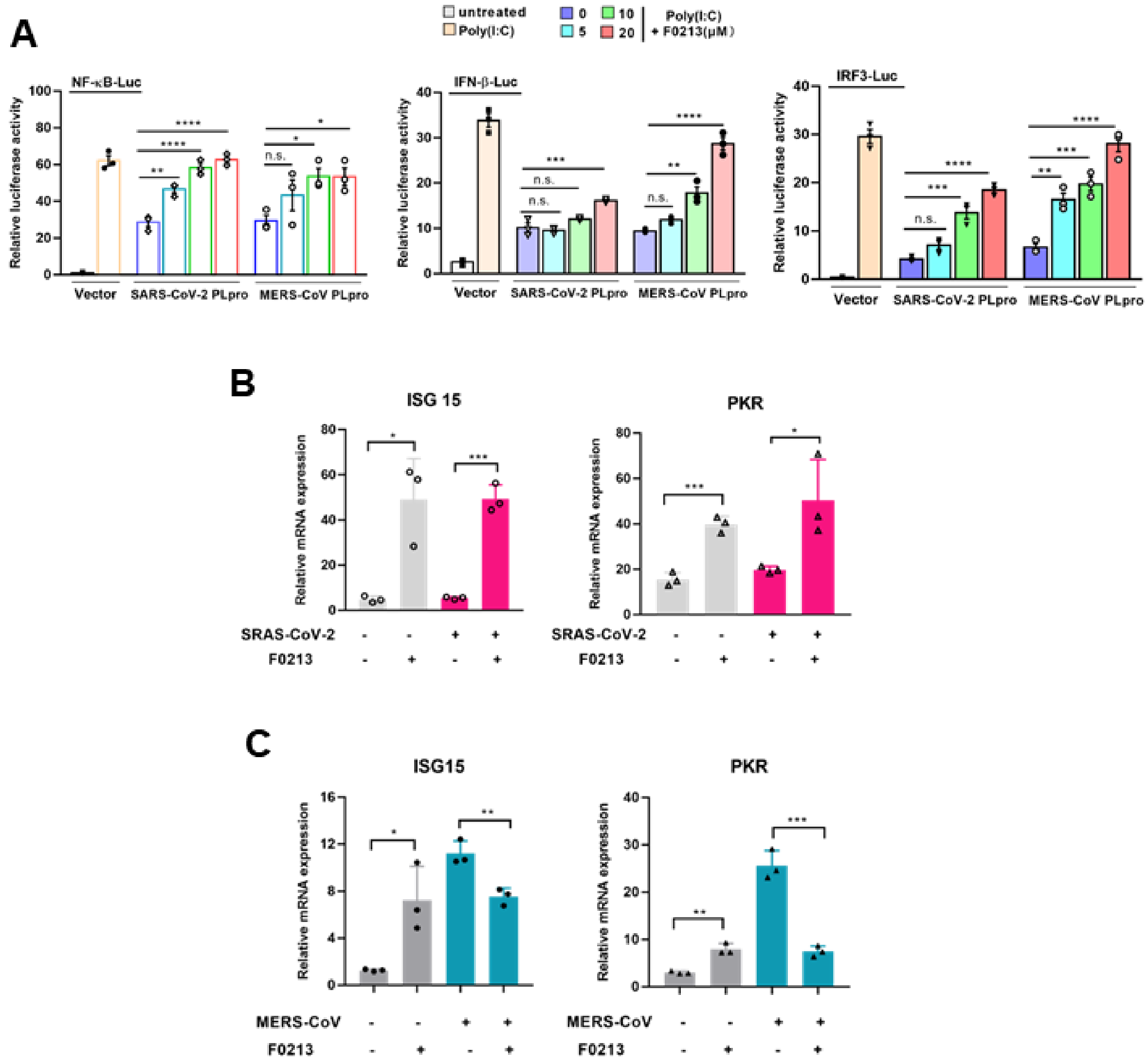

Figure S3. F0213 antagonizes PLpro-mediated immune dysregulation. (A) F0213 antagonized PLpro suppression on NF-κB or IFN-β or IRF3 expression. Dual-luciferase reporter gene assays were performed in HEK293T cells. Cells were transfected with indicated SARS2-PLpro or MERS-PLpro and treated with poly(I:C) to induce reporter gene expression, respectively. All data are presented as mean ±SD. One-way AVONA for statistical analysis were compared with the DMSO group (0 μM). For all statistical analysis, ****p<0.0001, ***p<0.001, **p<0.01, *p<0.05 and n.s. non-significant. (B and C) mRNA expression of IFN-responsive host genes in the presence of virus infection and F0213 treatment. RT-qPCR analysis was performed utilizing the cell lysate RNA extraction of Caco-2 (SARS-CoV-2, 0.1MOI, 24hpi) or Huh-7 (MERS-CoV, 0.1 MOI, 24hpi), with or without 10 μM F0213 treatment. Data was shown as mean ± SD (gene copy per 1000 β-actin). Student's T-test. *p<0.05, **p<0.01 and ***p<0.001.

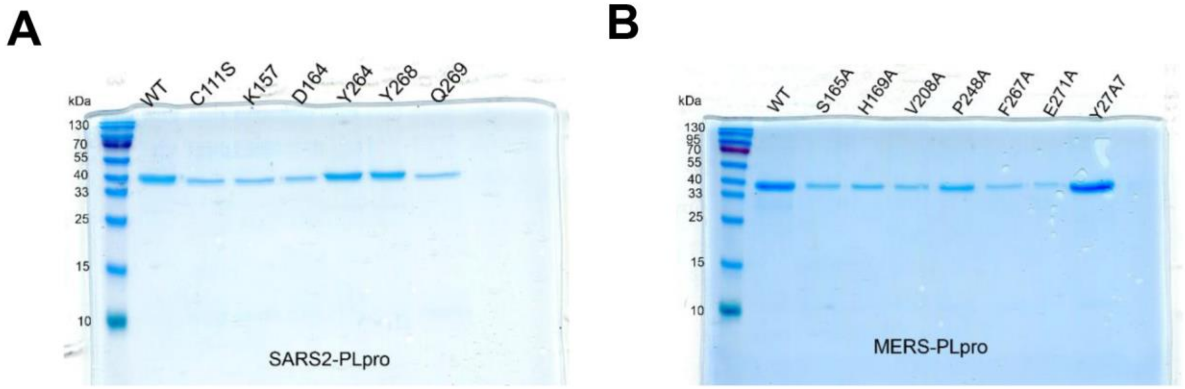

Figure S4. SARS2-PLpros and MERS-PLpro used in this study. (A) SDS-PAGE gel image of wild type (WT) and mutant SARS2-PLpro used in this study. (B) SDS-PAGE gel image of WT and mutant MERS-PLpro used in this study.
